# Pathogenic Exploitation of Lymphatic Vessels

**DOI:** 10.3390/cells11060979

**Published:** 2022-03-12

**Authors:** Alexandra I. Magold, Melody A. Swartz

**Affiliations:** 1 Pritzker School for Molecular Engineering, University of Chicago, Chicago, IL 60637, USA; melodyswartz@uchicago.edu; 2 Ben May Department for Cancer Research, University of Chicago, Chicago, IL 60637, USA

**Keywords:** lymph node, dissemination, replicative niche, enteric escape

## Abstract

Lymphatic vessels provide a critical line of communication between peripheral tissues and their draining lymph nodes, which is necessary for robust immune responses against infectious agents. At the same time, lymphatics help shape the nature and kinetics of immune responses to ensure resolution, limit tissue damage, and prevent autoimmune responses. A variety of pathogens have developed strategies to exploit these functions, from multicellular organisms like nematodes to bacteria, viruses, and prions. While lymphatic vessels serve as transport routes for the dissemination of many pathogens, their hypoxic and immune-suppressive environments can provide survival niches for others. Lymphatics can be exploited as perineural niches, for inter-organ distribution among highly motile carrier cells, as effective replicative niches, and as alternative routes in response to therapy. Recent studies have broadened our understanding of lymphatic involvement in pathogenic spread to include a wider range of pathogens, as well as new mechanisms of exploitation, which we summarize here.

## 1. Introduction

Lymphatic vessels drain interstitial fluid from most tissues and drive the interstitial fluid flow that carries proteins and macromolecules out of the tissue. Importantly, this provides the draining lymph nodes (LNs) with a constant stream of information about the specific tissues they drain, including information carried by immune cells that use lymphatic vessels for transport to the LN. Therefore, lymphatic vessels are not only considered important to the circulatory system, but are also important to the immune system. First, dendritic cells (DCs) and other antigen-presenting cells enter local lymphatic vessels to carry specific antigenic information to the LN for lymphocyte education; innate immune cells like neutrophils also use lymphatics for tissue exit [1,2]. Soluble factors also provide information that can affect immune responses, including antigens, cytokines, and immune-modulating micro ribonucleic acid (miRNA), which are often packaged into exosomes [3,4,5]. Second, lymphatic endothelial cells (LECs) themselves can directly modulate immune cells, including macrophages, DCs, and T cells, mostly in roles related to immune regulation [6]. Such regulation may be critical for both resolving effector responses and establishing memory, as well as avoiding autoimmune responses against autoantigens. Thus, as controllers of cells and information flow to the LN, and as important sensors and regulators of immunity, lymphatics are central to both orchestrating and regulating immune responses.

Paradoxically, while these functions evolved to efficiently and appropriately fight infection while preventing autoimmune reactions, many pathogens have developed ways to exploit them for their survival and spread. Although the involvement or exploitation of lymphatics has only been specifically explored for a small subset of known pathogens, examples from nearly every class of pathogen can be found, including viruses, bacteria, protozoans, prions, and even relatively large multicellular pathogens (helminths). For some, lymphatics can be critical routes for transport out of the site of infection, where they can infect other cells in the LN or enter the bloodstream to infect another tissue or organ that can serve as a niche for survival or replication. Others have evolved mechanisms to infect LECs or exploit their immunoregulatory functions. Finally, LECs thrive in hypoxic conditions [7,8], another feature that can be exploited by some pathogens.

Here, we review the evidence for the multitude of ways that pathogens may exploit lymphatic vessels for survival, dissemination, and replication. We summarize the strategic advantages of lymphatic dissemination in terms of the migratory immune cells with which lymphatic endothelia have prolonged contact, the immune suppressive environments that can be created, and the advantages of lymphatic vessels, and even LECs themselves, for replication. We also outline the pathogenic exploitation of lymphatics of the Peyer’s patches and lymph nodes.

## 2. Lymphatic Vessels as Escape Routes

Most pathogens enter the host after breaching an epithelial barrier. This breach can be accomplished indirectly (e.g., infection of mucus-patrolling macrophages [9]), directly (e.g., infection of inhaled SARS-CoV-2 virus by pulmonary epithelial cells [10]), or facilitated by a vector (e.g., intradermal ‘injection’ of malaria-causing *Plasmodium* by a mosquito [11] or a wound from a contaminated object (e.g., with tetanus-causing *Clostridium tetani*). Lymphatic vessels provide a route for pathogens to escape this entry point and move to a more favorable site or to disseminate more broadly. In this section, we highlight examples of pathogens that exploit vulnerabilities present in homeostatic lymphatic behavior to escape beyond their primary site of entry.

### 2.1. Escape from the Skin

Most insect-transmitted pathogens are introduced during a blood meal into the dermis, where patrolling phagocytic cells are abundant and lymphatic capillary density is high. Among the largest pathogenic organisms recognized to exploit the lymphatic vessels for survival are species of filarial nematodes that can cause severe lymphedema or elephantiasis, including *Brugia malayi*, *Brugia timori*, and *Wuchereria bancrofti*. These are introduced intradermally in the infective L3 larval stage from a bite by infected mites or mosquitos, after which they must enter lymphatic vessels rapidly or die in situ [12]. The reproductive worms can live up to 15 years in lymphatic vessels, where they not only impair lymphatic contractility and eventually block lymph flow, but also can support opportunistic bacterial infections that further drive lymphedema [13]. Recent studies have demonstrated that shortly after inoculation, they enter small collecting vessels that are supported by enough basement membrane to provide adequate stiffness for the larvae to dig an opening for entry into the endothelium [12]. Beyond the entry, the mechanisms that support long-term survival of these lymphotropic filariae in lymphatic vessels are incompletely understood, although the larvae can promote an immune-suppressive environment within the lymphatics by inducing host expression of IL-10 [14], a survival mechanism also exploited by *Leishmania* [15], *Yersinia pestis* [16] and *dengue virus* [17].

Unlike filaria, with muscle cells that can mechanically disrupt the lymphatic wall [12], most other vector-borne pathogens do not have the mechanisms to actively enter into a lymphatic vessel. Still, several reports have documented observations of free pathogens inside lymphatics (mostly from intradermally injected pathogens), including infections of *Y. pestis* [18], *Streptococcus pyogenes* [19], and *Salmonella* [20]. Classical electron microscopy studies by Leak [21], Casley-Smith [22], and Schmid-Schonbein [21] have examined the overlapping cell–cell junctions of lymphatics and noted that, where gaps exist, they are typically smaller than ~100 nm [22,23], but that larger gaps in the order of ~1 μm can occasionally be found; thus, it is possible that pathogens like the immotile, large (~1 μm) *Y. pestis* may freely flow into lymphatic vessels across such gaps. On the other hand, lymphatic endothelial cells are rich in caveolae that engulf solutes, and transcytosis is an important route for translymphatic solute transport [22,23,24], so this may be another means by which some pathogens gain direct entry.

Besides direct entry, pathogens often exploit migratory immune cells like DCs [25], macrophages [26], and neutrophils [27], which can be rapidly recruited to the site of inoculation. Upon entry via phagocytosis or direct infection, the pathogen manages to evade the phagocyte’s usual destruction mechanisms (e.g., proteolytic degradation and acidification within endosomes) without killing the cell immediately, thus allowing it to hitch a ride as the cell migrates to the draining LN and other distant organs [25,28,29].

**Figure 1:**
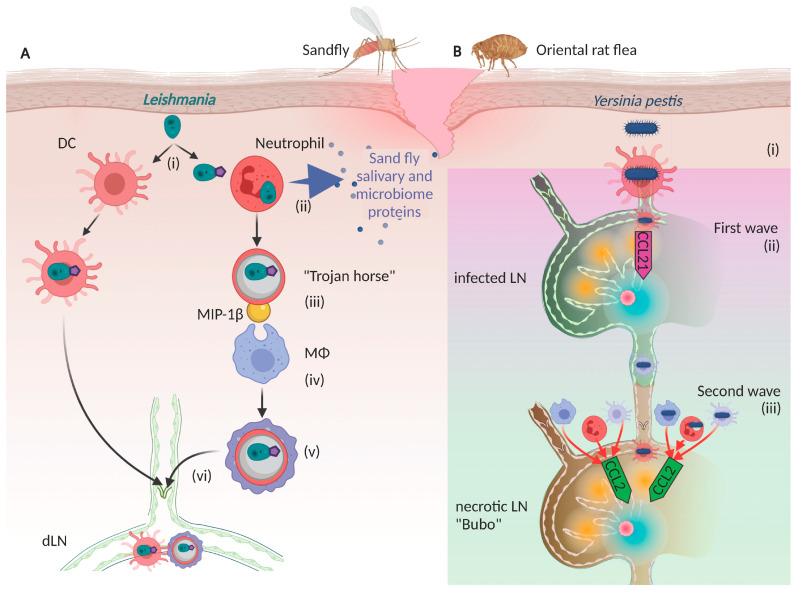
Lymphatic escape routes of skin infections, (**A**). *Leishmania* is introduced by the bite of a sand fly, where (i) its unflagellated (amastigote) form infects neutrophils and dendritic cells (DC). (ii) Neutrophils are attracted to the infection site by chemokine-like proteins of the sandfly microbiome and non-chemokine-like yellow proteins of the sandfly saliva. (iii) Amastigotes are coated with lipophosphoglycan (LPG, purple pentagon), which prevents phagolysosomal maturation and thus prolongs pathogen survival within neutrophils. (iv) LPG also induces macrophage inflammatory protein-1b (MIP-1b) expression, which attracts macrophages (MF) that (v) phagocytose the infected “Trojan horse” neutrophils. LPG also allows amastigotes to infect dendritic cells via toll-like receptor 2 (TLR2) mediated interactions (left route), and (vi) both types of infected cells enter lymphatic vessels to access draining lymph nodes (dLN), where infection propagates. (**B**). *Yersiniae pestis* is introduced by the bite of the Oriental rat flea, (i) where it is taken up by DCs. Infected DCs home to draining lymph nodes in small numbers, but (ii) the infection upregulates the lymphokine CCL21, which further attracts DCs to the LN, initiating the first wave of DC migration. (iii) As the infection progresses, CCL2 becomes upregulated, driving a second wave of DC migration along with consequent infiltration of large numbers of infected and non-infected permissible cells such as neutrophils, macrophages, and DCs. The influx of macrophages and other cells can be so extensive as to drive excessive LN swelling and necrosis, evolving into the characteristic “bubo” of bubonic plague.

The trypanosome *Leishmania* is one such parasite. It is mostly transmitted in its amastigote (unflagellated or immotile) form by the bite of an infected sandfly (Figure 1). Sandfly saliva contains proteins from both saliva and the microbiome, which can inhibit complement-mediated clearance [30], as well as directly or indirectly recruit phagocytotic cells and neutrophils [31] via the induction of IL-8 in endothelial cells [32]. Macrophages are its main cellular reservoir [33,34], but it first infects neutrophils that are attracted to the site as an intermediary [35]. The amastigote parasite secretes lipophosphoglycan (LPG), which prevents lysosomal maturation (and thus the destruction of the pathogen) and induces expression of Macrophage Inflammatory Protein-1b (MIP-1b) to attract macrophages and provoke neutrophil uptake by macrophages. In this way, the pathogen-infected neutrophil becomes a “Trojan horse”stratagem to infect macrophages [27,30,36]. Once inside macrophages, the LPG can trigger expression of Hypoxia-inducible factor 1 alpha (HIF-1a), in turn triggering local expression of vascular endothelial growth factor A (VEGF-A) to drive local lymphangiogenesis [37].

In another example, *Yersinia pestis* is a lymphotropic, gram-negative bacteria transmitted to the skin by the bite of an infected Oriental rat flea. This bacteria was responsible for the bubonic plague, a disease characterized by the ‘bubos’ or infected, necrotic LNs that are excessively swollen. These bubos can develop as a consequence of two consecutive waves of infected DCs [25] (Figure 1B). First, *Y. pestis* infects DCs at the infection site, which migrates into lymphatic vessels and to the draining LN in small numbers, representing the founder population. This is mediated in part by upregulation of the lymphokine CCL21, and it was shown that a CCL21 blockade could prevent this first wave of DCs, in turn slowing the spread of *Y. pestis* [25,38]. From there, increased LN expression of Sphingosine-1-phosphate receptor 1 (S1P1) may allow DC to exit and further spread the bacterium into the next downstream LN. In a second wave, the pathogen increases CCL2 expression to motivate the influx of more macrophages, neutrophils, and even DCs, transforming the secondary downstream LN into the aforementioned bubo. It has been suggested that S1P1 expression is important for this second wave of infection, as blocking S1P1 to prevent DC egress from the first LN was effective in halting disease progression in infected mice [25,38].

*Dengue virus*, which causes hemorrhagic fever as a consequence of antibody-dependent enhancement response following secondary infection [39], also enters the skin via the bite of an infected mosquito, where it mainly infects Langerhans cells [40] but also neutrophils [41] that traffic to the LN. There, infected cells localize to the subcapsular and medullary sinuses [42], and its viral proteins elicit strong germinal center responses but suboptimal non-neutralizing antibodies [43].

These examples of helminth, nematode, bacterial, and viral infections demonstrate how co-evolution has allowed for pathogens to exploit lymphatic vessels. Specific exploitation can occur directly, where pathogens are being transported freely by lymphatic fluid that flows inside lymphatic vessels and disseminates the pathogens across the system to various extents, depending on the effectiveness of lymph node entrapment [19,44]. The spread can also happen indirectly, where pathogenic dissemination is mediated via host cells that carry the pathogen from the initial site of infection into the lymphatic system [45]. In the latter cases, the pathogens benefit from the sensing of chemotactic gradients to home to lymphatic vessels (with the exception of helminths) of their host cells. In cases where cell-mediated dissemination represents the dominant route, blocking immune cell trafficking might be a rational strategy. Promising results from mouse experiments have, for example, been published for the case of *Y. pestis*, where CCL21 blockade in early disease or S1P/S1PR1 inhibitors in later disease can interrupt the lymphatic spread of the pathogen [25].

### 2.2. Lymphatic Vessels as Escape Routes from the Gut via Peyer’s Patches

Unless ingested pathogens act on targets in the gastrointestinal lining, they must overcome an inhospitable environment, from acidic gastric pH levels [46], a multitude of digestive enzymes designed to break down proteins, fats, and carbohydrates, and also mucosal antibodies [47]. Mesenteric escape is, therefore, uncommon, and a major barrier exists in the form of a dense epithelial layer, reinforced by impregnable tight junctions that keep almost all commensal microbes and pathogens in the mesentery [48]. Under healthy physiologic conditions, all nutrient and solute uptake occurs via transcellular diffusion through the epithelium and underlying lamina propria, into the bloodstream [49].

**Figure 2:**
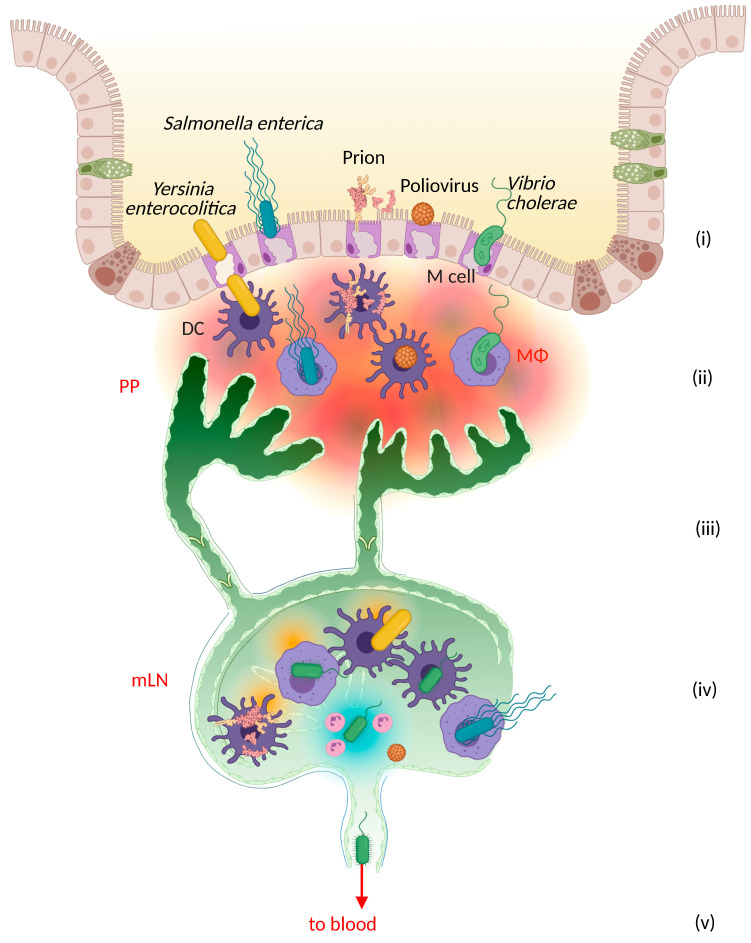
Enteric escape of ingested pathogens. Lymphatic vessels present opportune routes of escape from the intestinal lumen for pathogens such as *Yersinia, Salmonella*, *Vibrio cholerae*, *Poliovirus*, and *prions*, which favor microfold cell (M cell) shuttling to breach the epithelial barrier. (**i**) M cells shuttle pathogens across the follicle-associated epithelial dome above the Peyer’s patches. (**ii**) Patrolling dendritic cells, macrophages and other phagocytic cells become infected upon pathogen uptake, yet still (**iii**) enter lymphatic vessels and (**iv**) transmit the infection to the mesenteric lymph nodes (mLN). There, bacteria may exit the LN and (**v**) access the blood circulation via the thoracic duct.

Many pathogens share a preference for lymphatics to escape from the intestinal lumen and its underlying lamina propria [20,50,51,52,53,54,55,56,57,58,59]. Similar to pathogenic behavior during skin infections, enteric pathogens are often spread by riding inside surveilling immune cells such as DCs [60,61,62]. In contrast to the skin, however, the gut-associated lymphatic system consists of specialized sampling wall cells functioning similarly to afferent lymphatic capillaries, in the sense that they allow for antigen exposure inside the lymphatic structure (Figure 2). These cells are the microfold or M cells, which are part of the follicle-associated epithelium and directly shuttle molecules and infectious agents to the underlying Peyer’s patches (PP) [63,64,65,66]. PP differ from LNs in that, even under steady-state conditions, they are constantly active, with well-formed germinal centers surveilling the active intestinal microbiome [59,63,67].

A broad range of pathogens exploits the broad transcytotic activities of M cells to access lymphoid tissues for dissemination [52,66]. Some well-known examples include *Salmonella enterica*, *Yersinia enterocolitica* [68,69], *Vibrio cholerae* [59,70], prions [71], and *Poliomyelitis virus* [56,72]. Ultimately, these pathogens may either flow freely in the lymph into the PP or gut-associated lymphatic tissue (GALT), or enter DCs, macrophages, or other cell types en route to the PP or GALTs. From here, pathogens continue their trip to downstream LNs and then on to the bloodstream. In the case of *Vibrio cholerae*, M cells deposit the bacteria with migratory macrophages and DCs en route to the PP [59,70]. In a *Salmonella* infection, half of the bacteria may travel freely in the lymph before being taken up by DCs, macrophages, and B cells in the mesenteric LNs [20].

However, this route is not always linear, as the host cells might skip the lymphatic vasculature entirely to access the blood. For example, DCs can serve as vehicles for *Salmonella* to navigate lymphatic vessels towards the mesenteric LNs [20], but the intracellular secretion of Salmonella plasmid virulence C (SpvC) can be used to direct DCs towards the blood endothelium [73], creating a faster route to systemic dissemination. In addition, *Salmonella* may also access CD18+ phagocytes, which are capable of directly sampling the gut lumen, to enter the blood, although these cells typically follow chemotactic gradients to the lymphatic system [74].

### 2.3. Lymphatic Vessels as Survival and Replicative Niches

The LECs that constitute lymphatic vessels can also be hosts for pathogen survival and replication. As gatekeepers for transport to the lymph nodes, LECs occupy positions of strategic advantage, due to their contact with transmigrating macrophages, DCs, neutrophils, and other migrating leukocytes. When LECs are infected with viruses such as human *Cytomegalovirus* (HCMV) [75], they can upregulate leukocyte adhesion receptors such as ICAM-1, e-selectin and VCAM-1 [76,77]. This, in turn, slows the transmigration of leukocytes and prolongs cell–cell contacts during transmigration, which allows for efficient viral transmission to the transmigrating cell [75]. In addition, the physical characteristics of lymphatic flow and their luminar environments may create permissive macroscopic niches wherein microbes may be incubated, such as what has been described below for some small colony variants of methicillin-resistant *Staphylococcus aureus* (MRSA) [44,78].

When LECs themselves become the targets of infection, they can serve as reservoirs for the local accumulation of pathogens that subsequently infect neighboring cells. In leprosy, for example, *Mycobacterium leprae* infection of Schwann cells leads to demyelination, causing peripheral nerve damage and neuropathy; however, how the pathogen could transverse the multiple layers of connective tissue surrounding the nerves is unclear. Two decades ago, it was suggested that nerve-adjacent lymphatic microvessels could harbor the pathogen, allowing it to accumulate before infecting neighboring Schwann cells [79]. In an armadillo model following intravascular inoculation with *M. leprae*, the investigators found extensive evidence of LEC infection, as well as intralymphatic LEC-bound bacteria. Furthermore, it was found that in *M. leprae*-infected cutaneous plaques in humans, damage to the microvasculature (including lymphatics) caused decreased tissue perfusion, potentially contributing further to the neuropathy [80,81].

This begs the question of whether there are any molecular features specific to LECs that may make themselves amenable to becoming a substrate for the growth of microbial pathogens. In one notable example, *Streptococcus pyogenes* (Group A Streptococcus), which spreads via lymphatics [19], possess hyaluronan-based capsules that are able to engage the LEC-specific receptor LYVE1 [82,83]. This mechanism has been suggested as an explanation of how occult infections might rapidly enter lymphatic vessels and subsequently disseminate throughout the body, facilitating bacteriaemia [19].

The herpes-causing human *Cytomegalovirus* (HCMV), is typically transferred by contact with bodily fluids, and infection of the upper respiratory tract is a dominant route of initial infection [84]. Specifically, it enters through the cilia of sensory olfactory neurons [85], which differ from many other cellular epithelial surfaces by the presence of a heparan sulfate layer, which the virus uses to enter the cell [86,87]. In addition to heparan binding, HCMV also uses neuropilin-2 (Nrp2) as an entry receptor [88,89,90]. Following this initial infection of olfactory neurons, viral dissemination occurs through infection of leukocytes and endothelial cells [75,77,85,88,91,92,93,94,95,96,97,98] in a symbiotic fashion; infection of endothelial cells drives increased vascular permeability, which enhances leukocyte transmigration, in turn increasing transmission from the endothelium to the migrating leukocyte [77,84,99]. Interestingly, LECs specifically express Nrp2 and heparan sulfate proteoglycans compared to blood endothelial cells [100,101], and thus it is likely that infection of the lymphatic endothelium may play an important role in viral dissemination.

**Figure 3:**
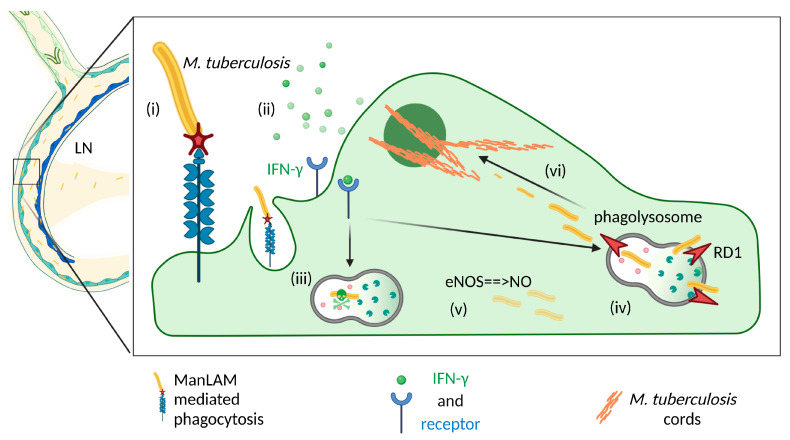
Lymphatic endothelial cells serve as replicative niches for extrapulmonary *Mycobacterium tuberculosis*. (**i**) *M. tuberculosis* is uptaken via mannosylated lipoarabinomannan (ManLAM)-mediated phagocytosis. (**ii**) If LECs are activated via IFNγ, the endocytosed bacteria can be killed via (**iii**) phagolysosomal degradation and (**iv**) eNOS-driven nitric oxide. (**v**) However, if RD1 is expressed, *M. tuberculosis* can escape from phagolysosomes into the cytosol, where (**vi**) it forms extensive cords that evade intracellular degradation and permit extensive replication, often almost filling the entire cytosol before killing the cell.

*Mycobacterium tuberculosis*, which is among the deadliest bacterial pathogens in humans, has always been described with lymphatic manifestations such as cervical lymphadenitis (scrofula) [102,103] or granulomatous thoracic lymph nodes, termed Ghon complexes [104,105]. While the bacterium mostly infects macrophages in the lungs, the extrapulmonary bacterium can infect LECs via their mannose receptor [106], contributing to its dissemination. An interesting feature of this pathogen is its ability to form long cords, whereby hundreds or thousands of bacteria are strung together [107]. Intracellularly, these form only after a subset of virulent bacteria expressing region of deletion 1 (RD1) manage to damage and then escape the phagosome. Subsequent rapid cording within the cytosol allows for the size-dependent evasion of cytosolic sensors and, thus, avoidance of clearance by intracellular destructive pathways such as xenophagy [107]. Eventually, the replicating bacteria exhaust intracellular nutrients and cause apoptosis of the infected cell, which releases them into the extracellular space where they block phagocytic uptake and continue to disseminate. Interestingly, recent evidence has demonstrated that LECs can survive with far higher burdens of cytosolic bacteria than can macrophages [107], where infection tends to induce necrotic cell death, suggesting that LECs may serve as particularly favorable replication niches for *M. Tuberculosis* (Figure 3).

Eventually, such infections can also directly impair lymphatic function, sometimes to the advantage of the pathogen itself. Following MRSA infections of LECs, the damage may impair lymphatic pump function, leading to persistent lymphedema, months after the initial infection has been cleared [78]. As MRSA may also infect the lymphatic vessel-associated smooth muscle cells, the resulting disorganization further impairs the recovery of vessel function [78]. This environment, with interrupted lymphatic flow and impaired local immune cell trafficking, creates a temporary niche wherein MRSA small-colony variants may continue to replicate, causing recurring MRSA infections [78].

Taken together, the environments within lymphatic vessels or their endothelial cells may be favorable environments for various pathogens to be incubated while they replicate. In doing so, they access high-traffic areas that are advantageous for easy access to, and frequent contact with, immune cells en route to downstream LNs, or alternatively, a compartment with low shear stresses compared to those in blood vessels, facilitating stable adhesion to the endothelial layer.

## 3. Conclusions

New developments continue to elucidate the roles of the lymphatic vasculature as a means for the escape, dissemination, and replication of many pathogens. Pathogens can exploit the unique hypoxic conditions under which LECs thrive, their location as ‘exit routes’ from sites of entry in the skin and gut, their physical contact with migrating immune cells to the lymph node, or their immune-suppressive features, among others. As a function of their location, LECs can be strategic spaces for distributing pathogens in general, and specifically for breaching physical barriers such as the connective perineural tissue or the epithelium and the lamina propria of the digestive tract, either to transmigrating mobile cells that can then carry pathogens downstream, or for replication and expansion of the pool of infective pathogens. In some of the diseases mentioned above, lymphatic-targeted interventions are viewed as a strategy to stop disease progression in its earlier stages, before dissemination and late-stage complications have a chance to develop.

## Data Availability

Not applicable.
